# Perceptions and experiences of adolescents, parents and school administrators regarding adolescent-parent communication on sexual and reproductive health issues in urban and rural Uganda

**DOI:** 10.1186/s12978-015-0099-3

**Published:** 2015-11-30

**Authors:** Wilson Winstons Muhwezi, Anne Ruhweza Katahoire, Cecily Banura, Herbert Mugooda, Doris Kwesiga, Sheri Bastien, Knut-Inge Klepp

**Affiliations:** Department of Psychiatry, Makerere University College of Health Sciences, School of Medicine, P. O. Box 7072, Kampala, Uganda; Child Health and Development Centre, Makerere University College of Health Sciences, School of Medicine, P. O. Box 7072, Kampala, Uganda; University of Calgary, Global Health & International Partnerships, Faculty of Medicine, 2500 University Drive NW, Calgary, AB T2N 1 N4 Canada; Institute of Basic Medical Sciences, Faculty of Medicine, University of Oslo, Oslo, Norway

**Keywords:** Parents, Adolescents, Uganda, Sexual and reproductive health, Sexuality communication, HIV prevention

## Abstract

**Background:**

Evidence suggests that in spite of some adolescents being sexually active, many parents do not discuss sex-related issues with them due to lack of age-appropriate respectful vocabulary and skills. The likelihood of parent-adolescent communication improving sexual and reproductive health outcomes appears plausible. The desire to understand parent-adolescent communication and how to improve it for promotion of healthy sexual behaviours inspired this research. The paper is meant to describe perceptions of adolescents, parents and school administrators about parent-adolescent communication on sexual issues; describe the content of such communication and identify factors that influence this communication.

**Methods:**

The study was done among two urban and two rural secondary school students in their second year of education. Data were collected from 11 focus group discussions and 10 key Informants Interviews. Data management, analysis and interpretation followed thematic analysis principles. Illuminating verbatim quotations are used to illustrate findings.

**Results:**

Parental warmth and acceptability of children was perceived by parents to be foundational for a healthy adolescent- parent communication. Perceptions of adolescents tended to point to more open and frequent communication with mothers than fathers and to cordial relationships with mothers. Fathers were perceived by adolescents to be strict, intimidating, unapproachable and unavailable. While adolescents tended to generally discuss sexual issues with mothers, male adolescents communicated less with anyone on sex, relationships and condoms. Much of the parent-adolescent communication was perceived to focus on sexually transmitted infections and body changes. Discussions of sex and dating with adolescents were perceived to be rare. Common triggers of sexuality discussions with female adolescents were; onset of menstruation and perceived abortion in the neighbourhood. Discussion with male adolescents, if it occurred was perceived to be triggered by parental suspicion of having female ‘friends’ or coming home late. Peers at school and mass media were perceived to the main source of sexuality information.

**Conclusions:**

Communication on sexuality issues between parents and their adolescent children was infrequent and critical elements like sex and specifics of protection against undesirable sexual behaviour consequences were avoided. Peers, schools and mass media should be creatively harnessed to improve parent-adolescent communication about sexuality issues.

## Background

Universally, consensus is emerging about the importance of improved parent-adolescent communication in promoting healthy sexual behaviours among adolescents [1–3]. Adolescents who have a positive relationship with their parents are known to be less likely to initiate sex early [3, 4]. There is growing evidence showing that various parenting dimensions like connectedness, love, material support, behavioural control, monitoring, and parent-adolescent communication are positively associated with reduced levels of risk-taking among adolescents [3]. In a study where researchers used in-depth interviews among young people in South Africa, it was found that most of them had positive attitudes to parent-adolescent communication, wanted parents to talk about sex, but that discussions with parents on sexual behaviour topics were rare [5]. These findings suggest that in order to promote such communication, it may be necessary to address socio-cultural barriers [3]. The 10–14 age range is a time of change, vulnerability and opportunity for adolescents to learn and develop skills to help them build patterns of health-maintaining behaviours. It is a time when adolescent can best be protected from potential risks by parents or caregivers who are closely involved in their lives [4].

Literature from Uganda on the role of parent-adolescent communication in promoting healthy sexual behaviour among adolescents is scarce. Other than some attempts of *Sengas* (biological sisters of fathers) and *Kojjas* (biological brothers of fathers) [6–8], the little information available suggests that parents are ill-prepared for this task [9]. Like elsewhere in sub-Saharan Africa (SSA), parental discussions of sexuality issues with their children in Ugandan culture are taboo [3, 9, 10]. This task is often relegated to other family members notably, paternal aunties in Buganda [9]. In talking about HIV, parents are often known to communicate with their children through arousal of fear. Parents in Uganda are known to be strict, particularly with the girl child, which prompts many to hide their intimate or sexual relationships thereby exacerbating their vulnerability [11]. Knowledge about HIV transmission and prevention, pregnancy prevention and condoms seems to be very low among young adolescents [12, 13].

Parental inability to communicate with adolescent children is even more limited when the adolescents are HIV sero-positive. As a result, parents completely avoid discussions on sexuality with such adolescents, yet these adolescents are known to be sexually active [14]. Research that hat explored sexual behaviour and desires among perinatally infected with HIV adolescents aged 15–19 years in Uganda found that 33 % had already had sexual intercourse [14, 15]. These findings underscore the urgency of enabling parents to communicate not only with young adolescents that are HIV negative, but also those who are HIV positive, in order to prevent further spread of HIV, to avoid unwanted pregnancies and other associated problems.

More research found that adolescents receive information about sexual and reproductive health (SRH) issues from various sources including school, other adults, the media and friends [16, 17]. Some of this information may be incorrect. In most cases, young people seek information after they have become sexually active [18]. Adolescents’ preference for information sources maybe based on their level of knowledge and perception of sources’ ability to maintain confidentiality [19]. In spite of socio-cultural barriers to effective communication between parents and children on SRH issues, such as age and gender hierarchies, adolescents in Uganda view their parents as a key source of information. In a multi-country study in SSA, more than half (51 %) of the girls and 27 % of the boys indicated that their parents were a key source of information [12].

Schools are often promoted as cost-effective settings to reach large numbers of adolescent youth with information and skills concerning SRH. However, many teachers in schools are uncomfortable to talk to young people about condom use. Many schools have a concern that access to SRH information may encourage young people to engage in sex. This is contrary to research showing that if young people are allowed early and complete access to SRH education, they are more likely to take less risks when they eventually initiate sexual activity [20]. It could also be due to lack of confidence by teachers to provide SRH education and also due to fear of parental backlash [21, 22].

Documentation about SRH projects suggests paucity of comprehensive programmes to address SRH in secondary schools in Uganda. While there have been attempts to implement SRH in secondary schools, there is ineffective coordination, supervision, limited geographical coverage and plans to ensure sustainability plans [23]. In the secondary school curriculum, some aspects of SRH are such as HIV/AIDS and puberty integrated and covered in different subjects, though without clearly specified modules [23].

In addition, evaluation of HIV/AIDS in the Ugandan secondary school curriculum revealed that HIV/AIDS education was neither examinable nor required. Integration of SRH in secondary school curriculum was not only slow, but difficult to implement in privately owned secondary schools [24]. The conclusion was that delivery of SRH information and services in secondary schools was inadequate and un-standardized in terms of issues, message design, delivery and specific services offered [24].

Contrary to assumptions that younger adolescents aged 12 to 14 years are sexually naïve, evidence suggests that some of are sexually active [4, 12, 25]. However, such adolescents are often missed in surveys on knowledge and behaviour about SRH because of their young age [4, 26]. Consequently, they may not be reached by national programmes. This may leave them at a risk of sexually transmitted infections (STIs), unplanned pregnancies and other associated problems. Furthermore, sexually active young adolescents may experience embarrassment and find SRH services inaccessible. This potentially exposes them to an elevated risk of STIs. A 2002 study by African Youth Alliance in 24 Ugandan districts approximated that 5 % of 10–14 year olds had ever contracted an STI (6 % of females and 4 % of males) [25]. Other studies show that adolescents are aware of HIV, STIs, contraception and pregnancy. However, they lack in-depth knowledge that can enable them to make decisions [12, 25]. Increased and improved discussions with their parents about SRH issues could help them to develop a more in-depth understanding and may lead them to adopt healthier lifestyles. Of the few interventions on adolescent SRH in Uganda, the majority are school-based, which fails to adequately leverage the important contribution parents can make. Though some school-based have a parental component, they do not have evidence-based components which integrates parents.

Parents in much of Sub-Saharan Africa argue that they are not comfortable discussing sex-related issues with their children and lack an appropriate language, information and skills to communicate effectively on these particular topics [3]. UNAIDS advises that it is essential to intervene early before adolescents become sexually active. Understanding the nature of parent-adolescent communication on SRH and how best it could be improved to promote healthy sexual behaviour was the main objective behind the formative research that informed this paper.

This paper came out of formative research to explore parent–child communication on SRH; in preparation for one of the components of a larger project titled: *‘Promoting sexual and reproductive health among adolescents in Southern and Eastern Africa (PREPARE) through mobilization of schools, parents and communities’* implemented in Uganda*.* The main aim of PREPARE was to develop new and innovative programmes for the promotion of healthy sexual practices among young adolescents using schools as a gateway to delivery. The research focus for the Ugandan study was rolled out in three phases: (i) a formative and exploratory study, (ii) use of formative study findings to design an intervention for promotion and improvement of the quality and frequency of parent-adolescent communication on SRH and (iii) implementation and evaluation of the intervention. Specifically, this paper reports findings from the formative research in which we examined; (i) adolescents and parents’ perceptions regarding parent-adolescent communication on SRH issues, (ii) school administrators’ perceptions about parent-adolescent communication on SRH issues, (iii) the content of parent-adolescent communication on SRH, and (iv) identify factors that influence this parent-adolescent communication on SRH issues.

## Methods

### Study sites and context

Participants in this study were students and their parents from 4 secondary schools in urban and rural Uganda. For students, the median age for male (range 12–20 years) and female (range 12–19 years) participants was 15 years. Schools were a gateway to recruiting adolescents’ parents. Participating schools were those that served people in the low income stratum of Ugandan society. Two of the schools were serving a cosmopolitan urban population of Kampala while the other two were in rural Wakiso district. The intention was to purposively include 4 schools that captured perspectives of students and parents from different physical locations socio-economic backgrounds and gender. The rationale for choosing schools where students were non-residents was the likelihood of a high frequency of contact and interaction between students and their parents in such day-schools. Inclusion of the 4 schools enabled comparison across different strata.

Being the capital city of Uganda, the population in Kampala at the time of the study is heterogeneous with many ethnic and linguistic groups. However, Luganda and English are the most commonly spoken languages. Conversely, Wakiso has a largely rural population. The most commonly spoken language in Wakiso districts is Luganda. Other languages are spoken by most parents that either migrate or intermarry within the two districts. To a large extent, the adolescents in the age-range for this study spoke Luganda and English.

### Design of the study

This was an exploratory and qualitative study designed to elucidate how adolescents, parents, teachers and administrators in Uganda perceived parent-adolescent communication on SRH issues. The qualitative approach was appropriate for a number of reasons. The approach enabled elaboration of how students perceived SRH and communication with their parents; development of hypotheses for testing during implementation of the intervention; understanding of perceptions that influenced parent-adolescent discussion of SRH issues; captured the language and imagery of adolescents, parents and schools administrators; generated ideas for improvements of parent-adolescent communication.

In this study, a parent was defined in a broad way as a biological parent or a guardian who lived with and took care of the adolescent. To be included in study, student participants had to be non-residents and in their second year of secondary school. The selection criterion for a parent to participate was having a child in senior 2 in the study school. To be eligible to be in the study, students had to give informed written assent while parents had to give written informed consent. The exclusion criteria were being a student or a parent of a student in a privately-run secondary school. The reason for this was that dynamics of parent–child interaction with school dministration in private schools could be different from government-aided schools.

### Study participants and procedures

Data were collected between September and November 2010 from purposive samples of 11 Focus Group Discussions (FGDs) and 10 Key Informants (KIs) spread across study schools. There were 4 FGDs of male students, with a total of 56 participants, those of female students were 4 with a total of 63 participants, those of male parents were 2 (1 with 5 and another with 7 participants) and 1 FGD of female parents with 8 participants. There were 10 Key Informants (KIs) who were schools administrators and teachers. These were the *‘natural observers’* often interested in the behavior of adolescents around them [27]. Selection was done in such a way that it represented variation in the phenomenon of interest. To attain maximum variation, heterogeneity of participants was ensured through selection which adhered to diversity in age and gender differences of participants.

Participating parents were invited through their children to convene at secondary schools of their children to be recruited into the study. Moderation of FGDs and note-taking was conducted by trained and youthful male and female research assistants who were seamlessly acceptable to targeted adolescents. This was to assure FGD participants of the necessary comfort to relate and discuss. Male research assistants moderated FGDs comprised of male students and females did the same for FGDs of females. To avoid disruption of schools schedules, FGDs with students were conducted during lunchtime.

Before data collection, participants were told about the purpose of the study. Data were collected and analysed to a point where no more new information to enrich theme identification was forthcoming [28]. Theme identification started during the literature review and continued as long as patterns that captured interesting issues were emerging [29, 30]. For comparison, two coders started to notice and look for patterns of meaning and issues of interest like the ‘the accepting and loving nature of parents’, ‘adolescents’ perspectives about communicating with parents’, ‘timing of communication with young adolescents’, ‘adolescents’ communication with their parents’, ‘content of young adolescent-parent communication’, ‘topics normally discussed during young adolescent-parent communication’, ‘comfort in discussing SRH issues’, ‘mode of communicating SRH information’, ‘satisfaction with communication about SRH’, ‘sources of information about SRH for young adolescents’, ‘frequency of communication about SRH between young adolescents and their parents’, ‘factors limiting young adolescent-parent communication on SRH’ and many others as data collection progressed. The process of data collection stopped when we began to notice repetition of information–almost verbatim–from different study participants. The length of either FGDs or KIIs ranged from 60–120 min. In the data collection process, participants in FGDs were served either a soft drink or water. None were given monetary compensation since they lived adjacent to study schools (Table 1).

**Table 1:** Sample questions in data collection guides

Questions in students’ FGDs	Questions in parents’ FGDs	Questions in school managers’ KII guide
• What do you know about SRH?	• What do you know about sexual and reproductive health?	• Does this school offer sex education classes? (*probe: to which classes; how often; what time; length of a session; are teachers given prior training*)
• How do you access information (What sources of information do you use to find out) about SRH? (*probe for most common; most trusted*)	• What do you think about parents discussing SRH issues with their adolescent children? (*probe: especially the young ones, 12–14 years*)	• What topics are covered under this?
• What role do you think parents should play in adolescents’ SRH?	• Do you talk to your adolescent children about SRH issues? Why or why not? *(probe to find out if they talk to only one sex or both, the age at which the talks begin and how often)*	• What process is followed during these classes? (*is it the teacher giving information; other staff; how do students participate*)
• Do your parents ever talk to you about SRH issues? (*probe: at what age; which parent; how often; who starts the conversation; comfort*)	• [For those who discuss SRH issues with their adolescent children], what topics do you discuss with them? (*probe to find out how easy/hard it is to discuss these topics; which topics they consider a priority*)	• What methods do you use to encourage/ensure participation of parents in school activities? (*probe: success of these methods*)
• For those who discuss with their parents, what topics have you discussed with your parents? (*probe to find out most common*)	• How are these conversations held? (*probe: who starts it-parent or child; where?*)	• What is your opinion on parents discussing SRH issues with their adolescent children? (*probe: especially the 12–14 years old*)
• For those who do not discuss their parents about sexual and reproductive health, what could be the reasons for such?	• What challenges do you face in talking to your adolescent children about sex and reproductive health? (*probe: challenges talking to children of different sex*)	• What strategies do you think would be suitable to involve parents in discussing sexual and reproductive health issues with their children?
• What would wish to see or hear (content) from your parents about SRH discussions?	• What would make it easier for parents to discuss SRH issues with their children?	• Are there any other comments you wish to make or questions you wish to ask on the topic discussed?
• How are these messages passed on? (*as warnings, threats, lectures, discussions, etc.*)	• What do you think about schools providing SRH health information to your children?	
• Do you get satisfied/more knowledgeable after these discussions? Why or why not?	• What information would you want them to receive at school? (*probe: from who?*)	
• What are the challenges you face in discussing SRH issues?	• How can parents be encouraged to take part in school programmes on SRH issues?	
• What benefits have you gained from discussing sexual and reproductive health with your parents?	• Are there any other comments you wish to make or questions you wish to ask on the topic discussed?	
• What methods would you suggest to improve discussions on sexual and reproductive health with your parents?		
• What information on sexual and reproductive health would you like to receive from your parents?		
• Are there any other comments you wish to make or questions you wish to ask on the topic discussed?		

#### Focus group discussions (FGDs)

The FGD process and guide were conceived and developed based on FGD theory and the existing literature [31–33]. Unlike the FGDs of adolescents, the parents’ FGDs were conducted in *Luganda* given their modest literacy levels. The FGD guide was therefore translated into *Luganda* and back translated to *English* to ensure conceptual equivalence and cultural sensitivity. Any discrepancies between the original version and the back-translated version were discussed in a joint meeting of translators to come to a consensus on words or phrases to be used. For convenience, FGDs were conducted at schools. FGDs were useful in collecting data on attitudes, perceptions, beliefs and practices regarding parent-adolescent communication on SRH issues. Participation of students in FGDs oscillated between 5 to15 participants and they were designed to elicit rich data on sexuality communication.

#### Key informant interviews (KIIs)

Given their personal skills or position within schools, they were able to provide more information and a deeper insight since they were “natural observers” with interest in students around them. They were chosen because of their opportunity to observe student’s interaction [27]. We used this method to collect data from school teachers and administrators. The purpose of these interviews was to obtain in-depth information on the study phenomenon.

FGD and KII guides that were developed by the research team based on a review of the literature, field experience and research objectives guided data collection. Each guide had sections on; parent-adolescent communication, parent-adolescent communication on sexual and reproductive health, school programmes on sexual and reproductive health and school sex education as domains of inquiry The FGD guide for adolescents had sections on attitudes towards parent-adolescent communication on sexual and reproductive health, social influence and beliefs about SRH, self-efficacy, action plans and delaying sexual debut.

The research team discussed and reached a consensus on suitability of questions in the guides and pre-tested them on similar students in the same schools but these were excluded from the final study. Research assistants were trained on the following aspects of qualitative research: how to probe and paraphrase, and how to fulfill their ethical obligations. The purpose of training was to ensure that they developed ability to elicit exhaustive and in-depth data on study participants’ full story regarding parent-adolescent communication on SRH issues.

Each data collection session was tape-recorded after seeking permission of participants. Data was thereafter transcribed verbatim by a bilingual speaker following acceptable guidelines [29]. All research assistants took detailed field notes. Data collection was overseen by the principal investigator and supervisors. Each research team had a moderator and a note taker. Each member of the research team was required to keenly observe the context in the school setting and document salient features. This was necessary to back-up and contextualize data that was collected.

### Ethical considerations

We obtained ethical clearances from the Uganda National Council for Science and Technology Committee on the Study of Human Subjects and Western Norway Regional Committee for Medical and Health Research Ethics. We also received administrative clearance from school administrators. Student and their parents/guardians gave us written assent and consent respectively. We accorded study participants the necessary privacy and assured them of confidentiality about what they told us. We assured them of their liberty to freely withhold information if they were uncomfortable to give it.

### Data analysis

Analysis and data collection progressed hand-in-hand. Emerging themes and impressions guided data collection. Words, content and context of what was said by participants was analyzed to comprehend parent-adolescent communication on SRH. Data were analyzed and interpreted manually. The audiotapes of all the data collection processes were transcribed following standard guidelines [34, 35] into English, scrutinized, and categorized by a bilingual speaker. Transcripts were reviewed and checked against original audio recordings by a language expert to ensure translation accuracy. The transcribed data were compiled into a text document. The first author closely read each transcript several times to get familiar with the depth and breadth of data content, inscribe notes on margins of the data book, identify key words, search for more meanings and patterns, and write detailed notes on emerging themes [29, 30].

Each extract of transcribed data was subjected to thematic analysis [36]. Through constant comparison, emergent themes, sub-themes, and data extracts coded. Some tentative themes lacked data to support them; others could be accommodated in other themes, while others deserved to be broken down. Bearing in mind the objectives of the study, theory and literature; data content, study context and underlying clusters of concepts, and relationships between codes, themes, and different levels of themes were noted [30]. Thereafter, more review and refinement was conducted to ensure coherent patterns [29]. The final themes are discussed in the findings section. We used illuminating verbatim quotations from participants to illustrate major findings.

## Results

Analysis and interpretation of findings is based on nine broad themes. The outline of the themes is as follows: (a), Communication between parents and adolescents which has sub-sections on general adolescent-parent communication on SRH issues, adolescents’ view of communicating with parents about SRH issues and initiating a conversation about SRH issues; (b) content of adolescent-parent communication with subsections on adolescents’ as well as parents’ perspective of what is normally discussed; (c) comfort in discussing SRH issues; (d) mode of communicating SRH information, (e) adolescents’ satisfaction with communication about SRH with their parents; (f) frequency of communication on SRH between young adolescents and their parents; (g) sources of information about SRH for young adolescents; (h) drawbacks of adolescent-parent communication on SRH; and (i) strategies to improve adolescent-parent communication on SRH issues.

### Communication between parents and adolescents

#### General adolescent-parent communication on SRH issues

The predominant view among young adolescents in the study was that communicating with parents was deficient. Generally, they perceived parents to often be tough, harsh, fearsome and authoritarian. They also reported that most adolescents spend more time with mothers. Fathers were perceived to be more strict, intimidating, unapproachable and/or unavailable. In the event that fathers attempted a discussion with their adolescents, it was believed that they are more cordial with boys. Although young adolescents studied believed that it is good for parents to monitor, regulate and set behavioural limits for them, the consensus was on general parental harshness.

*“. . . we do not get on well with some of our mothers. They quarrel too much especially after they get into conflict with our fathers. They then turn all their anger on us* (FGD of girls in a rural school – giggling as they discussed)*.*

*. . . some of us who live with grandmothers find them to be very, very, very tough. They behave like soldiers. . . . If they see you talking to a male neighbour, they begin asking tough questions like: ‘who was that person? ‘what were you talking about?’ and so on* (FGD of girls in an urban school).

According to some school administrators, communication on SRH issues between parents and their children existed, albeit with many challenges. The concern was that parents were often busy to the point of failing to turn up at schools of their children when needed. Other school administrators were not sure whether parents had time to communicate with their children about SRH issues. One of the likely explanations was that many children were not living with biological parents:

*I don’t think that parents of children in this school and the village ever discuss these things with their children. Maybe, some few try . . . many children live with grandparents who don’t care about telling grand children about sex . . . Others live with single parents who are interested in making money and may even marry off daughters to get money . . .* (School head teacher in the urban school).

Other administrators held a contrary view. For instance, some acknowledged that parents communicated with their children as illustrated by the following extract:

*I know that some parents do talk to their children. I have met some . . . I was counselling some children and later on, I met the parents, and they told me they had talked about the problem I was handling with their child. . . . I know that they talked . . . about sex and its disadvantages. Some parents use both discussion and threats, for instance ‘avoid sex and study hard’ and ‘if you dare get pregnant, don’t come back here’ . . .* (Head teacher, rural school).

Generally, school administrators were of the opinion that it was a minority of parents who talked to their children about SRH issues. They felt that parents ought to talk more with their children on SRH issues even when it was culturally challenging. They concurred that culturally, fathers were not expected to discuss SRH issues with daughters, but mothers could discuss with their sons and daughters. In addition, they noted that fathers often limited their conversations with their children to academic work and general behaviour.

#### Adolescents’ view of communicating with parents

Communication with fathers seemed to be rare. While mothers found it easier to communicate, adolescents admitted that they too find it harder to discuss SRH issues. Although some male adolescents’ attending urban schools reported that they communicate with their parents on any topic, others disagreed. Those who trusted their parents’ revealed that some parents were open and couldn’t spread rumours about their children’s problems. For instance, male adolescents were of the view that if they told their parents about their experience of a wet-dream, parents wouldn’t go talking about it with the neighbours.

Other young adolescents believed that their parents do not trust them. Their frustration in discussing SRH issues with fathers is well illustrated by the following extract;

*“. . . communicating with our fathers is not easy . . . sometimes, we communicate via telephone and they advise us to take care of our young siblings because we are older . . . that is where the communication usually stops. . . it looks like our fathers do not think it is proper to discuss with them SRH matters. . .”* (FGD of boys in an urban school).

When asked about whom they felt comfortable to discuss with and challenges they face, both boys and girls found their mothers easier to freely and openly communicate with;

*“Our mothers always tell us about working hard in order to achieve our goals. Sometimes . ., they take us to help in their businesses. We normally talk when we are at their work places . . . they give us advice about relationships. They say that we should avoid relationships with girls because they can distract us and we lose focus in our academics”* (FGD of boys in a rural school).

*We talk to our mothers because we spend most of our time with them. Our fathers come home late and they are always too serious. When our fathers talk to us, they asks questions about our studies and that is all* (FGD of girls in a rural school).

#### Initiating a conversation about SRH issues

The main triggers for SRH discussions between adolescents and their parents included; the fate of friends that were victims of early sexual activity, delay in onset of secondary sexual features, parental perception of disobedience in the adolescent and adolescents’ adopting an ‘inquisitive’ stance. A common trigger to start conversations about SRH with parents for girls in the rural schools was when a girl in a comparable age-group would become pregnant. In such a case, parents would be reacting to a situation that occurred to another person. For boys, the commonest trigger for attempts to converse with their parents about SRH issues was the overt realisation that they were starting to associate with female friends:

“. . . *our parents started telling us about sex issues when we were about 12–14 years of age. Our mothers started telling us about these issues because they see us moving in the company of girls in the village. They tell us that our voices have started to be deep and we should not engage in sex with girls but to just be friends with them* (FGD of boys in a rural secondary school)*.*

On the appropriate age for initiating communication with young adolescents, mothers believed that talking with girl children should be done earlier because they mature faster than boys. They expressed a fear that much of the information that girls get exposed to could be incorrect. They felt that children aged 12–14 years were too young to be told about SRH issues. They feared that starting such a discussion could prompt them to engage in risky sexual behaviours. Some of the mothers suggested that parents should start discussing SRH issues with their young adolescents because of the environment in which they lived:

*We parents have spoilt our children. We begin showing them that mummy and daddy share a bed at an early age. Therefore I think 8 years is the best age for them to be told about sexual issues. . . I agree with 8 years because these days, children are exposed to various circumstances such as drunken fathers who come home drunk and begin mature talk in the children’s presence . . . with the mizigo* [small temporary houses in slums] *all around us these days, it’s better for parents to begin such talk with their children at about 8 to 10 years. . . because I sometimes hear those young people conversing how they heard their parents in action during the night . . . which means that at that age, they have started comprehending whatever takes place* (FGD 2, mothers caregivers).

School administrators noted that most parents wait for their children to become adolescents before they begin any discussions on SRH issues, which would be too late. Because of the exposure to sex in the media from an early age, school administrators felt that students need a conversation on SRH issues with their parents early enough:

*These are difficult times, with children are getting exposed to televisions, novels, movies and phones. Someone can have a phone on which they have stored all sorts of dirty pictures. It is therefore necessary to discuss issues of sex and reproductive health early, before children come to secondary school, unlike long ago.* (Teacher, rural school).

For school administrators and teachers, the conversations between parents and their children on SRH issues should start as soon as children become aware of their sexuality and body parts, which would be around 7 years. They believed that waiting for adolescence is too late since many children would have discovered many things.

### Content of adolescent-parent communication

#### Adolescents’ perspective of topics normally discussed

Adolescents in the study reported that there were topics they preferred to discuss with their mothers and not fathers. Adolescent girls preferred to confide in their mothers when it came to talking about body changes during puberty. They believed that mothers were better suited for this task because they had had similar life experiences;

*“Unlike fathers, mothers know about menstruation and other women’s things . . . so it is easier to talk to them”* (FGD of girls in a rural secondary school).

When it came to discussing dating and relationships, both boys and girls seemed to be comfortable talking to other adults in their networks, notably brothers, sisters, in-laws or aunties. They never considered their parents to be well positioned to discuss such issues. Others even preferred to discuss dating and relationships with people they were not related to; notably friends, teachers and neighbours. Incidentally, they conceded that this had disadvantages as illustrated by the case of one boy;

“… *I was in primary five and about 14 years old when I first received information about SRH issues. I got the information from a woman in our village who was my friend. She told me that I should encourage and give hope to my mother by showing her that I can lure a girl into sex and have a girl- friend. She told me that I have to show my mother that I am not impotent and that this can only be done if I have sex with a girl. . .”* (Adolescent boy in a rural secondary School).

The content of communication between young adolescents and their parents tended to focus on avoiding people of the opposite sex, self-control and the call to avoid sexual acts. Secondly, adolescents indicated that messages from parents were often laced with abstinence, perceived defects of condoms (for instance, condoms have holes), timing of pregnancy and sexually transmitted infections:

*“. . . they tell us to abstain from sex and to take care of ourselves. They tell us to avoid girls, to avoid night parties and festivities and to avoid dances* (FGD of boys in a rural secondary school).

*Our parents tell us that all condoms . . . have holes and advise us not to use them. . that when we are about to have our menstrual periods, we should be more careful and avoid sex or else we become pregnant . . . that if we have sex at an early age, we can get diseases like gonorrhoea, syphilis and you can also get other complications. . .”* (FGD of girls in a rural secondary school).

Most messages given by parents to adolescents seemed to often be in line with Uganda’s strategy against HIV/AIDS since the emphasis was on abstinence, faithfulness in marriage and protected sex. In most cases, parents were known not to disaggregate information they discussed with their adolescents. Some of the boys indicated that their parents were proactive in advising them to protect themselves as they related to the opposite sex. To illustrate this claim is the narration of an adolescent from a rural school:

*“. . . my father found my condoms which I had been given at the health centre on my bed and touched them. . . I was scared but he told me not to fear . . . the other condoms I had, got lost when someone visited our home and took them from my suitcase by mistake. . . My father brought me more condoms. Then, some girls came to visit me and my father asked who they were and I told him that they were my classmates. Later on, I overheard him discussing me with my mother that. I liked girls. My father then brought more condoms and put them on my bed. . .”*

A few adolescents in urban schools admitted that their conversations with parents included topics about body changes in puberty. A predominant view was that adolescents found it easier to discuss topics like wet dreams, menstruation and reproduction with trusted aunties and uncles *(Sengas and Kojjas)* than their biological parents. With *Sengas*, comfort levels of adolescents discussing topical issues like elongating the labia, genital hygiene pains associated with childbirth and dangers of peer pressure were high.

*“. . . we do not converse about all sexual issues with our parents. We get most of the information from certain ‘Sengas’ in the village. The Sengas instruct girls about the practice of pulling their labia to increase their sexual sweetness . . . They say that a sexually sweet woman should produce moderate amounts of fluid during sex . . . .”* (FGD of adolescent girls in a rural school).

#### Parents’ perspective of content of discussions with adolescents

Generally, data from parents concerning discussion with adolescent children was devoid of SRH issues. Where SRH issues appeared, they were not explicit and tended to be shrouded in a broader set of discussions related to cleanliness and hygiene, education, morality, gardening, cookery, house-keeping and dress code of young girls. Some SRH issues initiated by parents and their adolescent children included; dangers of giving birth before the appropriate age, problems associated with premarital sex and body changes during puberty. Information about condoms, sex and dating tended to be missing.

Some parents wished they could hold discussions with their adolescent children about sexually transmitted infections, abstinence, condoms use, and puberty. However, some parents; notably fathers expressed their discomfort in communicating with their adolescents about such issues because of deficiencies in the vocabulary of native language dialects. It was difficult for some people to conjure non-vulgar words in native languages to substitute explicit sexual terms when talking to their adolescent children about SRH issues.

*Many of us fear to mention some words which leaves the children not informed on some sexual issues . . . we usually talk to the children when a problem has occurred and it is always in form of a warning or a reprimand”* (FGD of fathers).

*. . . we cannot mention sexual issues in the way they are supposed to be for fear or embarrassing ourselves and scandalizing the children . . . we use words like ‘katafali’*(brick) *to mean ‘bums’ while talking to the girls . . . communication to the children is also influenced by religion and culture* (FGD of fathers).

Generally, fathers were more comfortable discussing matters dealing with education but not SRH issues. They often talked about the need for children to have good behaviour and problems that could arise from dropping out of school.

### Comfort in discussing SRH issues

Young adolescents expressed comfort, contentment, confidence and hope of discussing SRH issues with their parents. A number of them reported that often times, they curiously look forward to such an opportunity to discuss with their parents. Though there were some exceptions, young adolescents believed that timidity, caginess, embarrassment and reluctance to discuss SRH issues was more of the parents’ problem. For instance, girls from a rural school said that they did not fear to discuss SRH with parents but when asked if they could tell their parents that it was time for them to get boyfriends, they were shocked and exclaimed… *Eh! No!* In some cases, some adolescents would be comfortable talking to one parent but not the other as illustrated in the quotation below:

*We feel shy when talking about sexual issues with our parents . . . when some of us started menstruation, we were so happy and went to tell our parents about it . . . However, some of the parents asked us about what we wanted them to do about such . . this was very embarrassing and some of us cried over it* (FGD of girls in an urban secondary school).

Consequently, some adolescent girls admitted that they would rather talk to their siblings about SRH issues instead of their parents. On the other hand, some parents indicated that they preferred to talk to their own children but they felt shy. There was a belief by some mothers that if they talked to their own children about sex-related issues, it could prompt them into experimenting with sex. Some mothers acknowledged that even if a child had started to have sexual intercourse, that child would not let the parent know about it.

*“The child can easily tell you stories about others but can’t tell you about herself or himself, and when you decide to talk to him or her about certain things, he/she shows you that what you are saying is old-fashioned . . . that he/she even knows better than you”* (FGD 1, mothers/female caregivers).

The fear of being asked embarrassing questions made some mothers uncomfortable to discuss SRH issues with their children:

*. . . sometimes we feel shy but we find that we must be straight and talk to our adolescent children about some things or else they will hear or read about them in a wrong way from another source . . . some of us feel shy but we are forced to do it, especially with those topics to do with relationships and sex . . . we usually feel shy but we have to ‘*kwekazza’ *(be brave) and communicate these issues to our children* (FGD 2, mothers/female caregivers).

### Mode of communicating SRH information

The most common modes of passing on SRH information to adolescents were; counselling, teaching, advising and conversations. This was reported more by girls than boys in both urban and rural schools as illustrated;

*We get information about SRH issues through counselling and discussions with parents. The parents warn us against engaging in sexual acts with girls saying most of them are sick* (FGD of adolescent boys in a rural secondary school)*.*

Other forms of passing on SRH messages to adolescents elicited in this research included; threats, intimidation, quarrels and abuses. This form of passing on SRH messages was reported more by adolescents in urban schools and it tended to be similar for both male and female adolescents as illustrated;

*We get SRH information through quarrels. For example, when a mother finds you standing with a girl, she waits for her to go away and then starts quarrelling and in the process, you pick something* (FGD of adolescent boys in a rural secondary school).

Some young adolescent girls from the urban schools reported that their parents discussed SRH issues with them during or after watching sexually-provocative movies or television programs.

*. . . for many of us, we watch Nigerian movies with our parents. In the movies, it is common for a boy to get a girl and they enjoy sex. When the girl in the movie gets pregnant, the boy abandons her on the pretext that his father told him that he had not reached the stage of impregnating a girl. When the girl went back home, she started falling sick, like that. When she was taken to the hospital and the doctor told the parents what had happened, both the parents died because of the shock caused by their daughter’s action. The man got an accident and when the woman was told, she died because of high blood pressure. We think that if we were the ones and if we engage in sex and we get such an experience, we could lose our parents. After watching such a movie with parents, the parents take time off to warn us that if we engage in sex, we know what can happen based on the message in the movie” *(FGD of adolescent girls in a rural secondary school).

Other sources of SRH information reported by young adolescents included; youth-friendly newspapers, educational movies, humorous songs, comical shows, jokes and stories; the internet and other people like teachers, health workers, siblings and counsellors.

### Adolescents’ satisfaction with communication of their parents about SRH issues

Other than in the rural-based schools, some adolescents felt that they were over-loaded with advice from their parents:

*“Sometimes, we feel over-loaded with what parents tell us . . . to the point of becoming bored. This is because, parents bring up something over and over and you get tired . . . also, some parents restrict watching television because they think it brings problems”* (FGD of adolescent boys in an urban secondary school).

On the contrary, there were some young adolescents who were satisfied with communication of their parents about SRH issues.

*“. . . we feel satisfied because we know that what our mothers tell us is right. We do not expect them to tell us something wrong . . . of course, they wouldn’t be able to tell us everything because they are human beings and they do not know everything. We expect to learn more as we grow older but we are satisfied with what they tell us and we ask whatever we want to ask”* (FGD of adolescent girls in an urban secondary school).

Adolescent girls in the urban secondary schools seemed to have faith and trust in their mothers, making a point that parents are trusted sources of information. With the exception of adolescent boys in the rural secondary school, some adolescent boys did communicate often with their parents. Both boys and girls said that their parents were often embarrassed and shy; not open and only gave clues yet the children would have wanted to know more from them as illustrated below:

*We find our parents not to be direct . . . they talk to us about issues of sexual and reproductive nature by passing through proverbs and at times, we can’t understand what they mean* (FGD of adolescent boys in a rural secondary school).

*Some of us really feel fearful and embarrassed when parents attempt to discuss sexual and reproductive health issues with us . . . we feel shy . . . some of us cannot say some words . . .* (FGD of adolescent boys in a rural secondary school).

The difficulty for parents to discuss SRH issues with their own children could be attributed to the culture in which parents were brought up. Many of the parents indicated that they were not told about SRH issues by their own parents too:

*. . . in our culture; it is common knowledge that mothers do not talk about those things with their children . . . when we adolescents, there was not a single day mothers told us about such things . . .*(FGD of mothers in a rural area).

### Frequency of communication on SRH between young adolescents and their parents

Mothers preferred frequent communication with girls compared to boys. They also found it easier to discuss more with older adolescent girls compared to the younger girls. The reason given was that older girls had more issues to talk about. Mothers admitted that as their children grow, the nature of communication changes. Incidentally, a number of mothers conceded that by the time they attempt to open up a discussion, they find that their adolescent children already know much from their peers. Other mothers reported that their discussion with young adolescents was often prompted by occurrence of a socially unacceptable problem like when someone else’s daughter would get pregnant. Many of the mothers reported that they couldn’t talk to their own children about using condoms. They were apprehensive and feared that discussions about condoms could give their young adolescents a false sense of protection and prompt them to have unprotected sex. They also felt that it was difficult for them to talk to their own children about other SRH issues:

*“. . . the truth is that we . . . have to summon all the courage to talk about SRH issues because it’s not easy sitting with a child you produced to tell him or her those issues. It is so hard . . . the child can even ask you what sexual intercourse is all about yet she or her knows what it is and besides, it is embarrassing”* (FGD of mothers in a rural area).

### Sources of information about SRH for young adolescents

In both urban and rural secondary schools and for both sexes, the most frequently mentioned source of information on SRH in the FGDs with adolescents was the school. Other than the science lessons in school, this was found to be from of documents like HIV/AIDS publications and science text books. Relevant school health clubs were known to provide related information as well. Whereas many young adolescents of both sexes got information from peers, there was an admission that some of this information was misleading as illustrated;

“. . . … *some friends say that using a condom during sex is like eating a sweet in its cover. Some friends also say that taking a pain killer like paracetamol tablets can prevent one from being found HIV positive if he/she takes an HIV test. . . Our friends also tell us that using family planning pills will make our ova to get spoilt”* (FGD of young adolescent girls in a rural secondary school).

Parents and other people at home were also cited as sources of information on SRH issues. Ultimately, the most common sources of information were; mothers, mass media and teachers. When asked what their preferred source of information was, the most frequently given response was mothers. Adolescents preferred less fear arousing communication from their parents. They revealed that their challenge was with parents who give them counsel in a rude manner, quarrel too much, threaten a lot and are scary.

*Some of our parents are too rude . . . sometimes, when you ask a question, the way they look at you scares you and you don’t feel comfortable to ask another question”* (FGD of young adolescent girls in an urban secondary school).

### *Drawbacks of* adolescent-parent communication on SRH issues

Timidity, shyness, embarrassment and fearfulness were found to be the main drawbacks for adolescents as they attempted to discuss SRH with their parents. Some feared to ask their parents to either avoid giving an impression that they were sexually active or to be misinterpreted and consequently punished. Others indicated that that their parents refused to answer their questions or asked them to go and ask their teachers or grandparents. Additional reasons given by young adolescents for not discussing SRH with their parents included fear of making parents angry, fear that parents would divulge their ‘secrets’ to neighbours and visitors, belief that parents were too busy and belief that parents were shy too. Parents admitted that they are often held-back from effective discussion of SRH issues with their young adolescents because of similar drawbacks. Some parents believed that such discussions would be uncomfortable for their children while other felt inadequate as illustrated;

*. . .the relationship with our children is good . . . however, regarding girl children, we fear talking to them direct issues related relationships especially things which are immoral . . . we mean conversations related to sex. Such things, we really fear to talk about them . . .* (FGD 1 of mothers).

*. . . most of the children are shy. . . especially the adolescent boys are shy with us when it comes to us mothers. They don’t reveal everything to us . . . so we basically rely on guesswork. . . . The girls are better, they are easy with us. But as they grow, they also begin the habit of silence as well. They don’t even want to mention when they begin their monthly periods* (FGD 2 of mothers).

Some adolescent children were known to block discussions about SRH issues with their parents as illustrated below;

*. . . for some children, when a parent tries to talk to them about HIV, they tell you that they learnt those things at school. In such a case, we as parents always insist on HIV and when we do so, they leave us there and walk away and this discourages many of us. For sure, we don’t normally talk to them because they show us that they do not want to know* (FGD 1 of mothers).

Mass media was reported to bombard children with information, some of which was misleading. For example, it was known that some television programs were passing on bad values, for instance explicit sexual innuendos to children. Parents believed that television, radio and newspaper programs rarely displayed culturally acceptable norms and values;

*. . . The films that are shown on television stations these days are doing much in spoiling our children. They do not know what programs to show at what time. You find that they are showing a movie that should have been shown after midnight because of its adult content during the day. . . the radios play more entertainment than educational programs . . some of the newspapers show pictures of naked people and our children are taken up by those scenes . . .* (FGD 2 of mothers).

### Strategies for improving adolescent-parent communication on SRH issues

Adolescents from urban schools suggested that parents should set aside time to talk to them in a ‘nice’ way. They also suggested that parents should try to express their love; encourage them to talk; pay attention to their views, guide them; try to understand them and be approachable as expressed in the following extracts;

*“Parents should create good relationships with their children. They should not always show them tough faces because it encourages their children to talk confidently. . . communication can be improved by parents encouraging their children to present their problems. . . this can help to remove the fear that they may be having”* (FGD of young adolescent girls in a rural secondary school).

Young adolescents also suggested that their parents should step-up their parenting skills. They believed that parents ought to encourage initiation of conversations on SRH issues and needed to desist from postponing responding to questions. Parents were also called upon to listen before reacting, to stop forcing their children into early marriages, to take their problematic adolescent children to professional counsellors for help, to stop telling their adolescent children that they themselves were not told anything when they were growing up, to organize themselves into groups to discuss how to be good parents, to attend school meetings and to talk more with teachers, and be to confident enough to talk.

Adolescents also believed that they had to be open; stop fearing their parents; initiate conversations; demand information; talk frankly; be confident and be friendly with parents. From the parents’ perspective, sensitization of parents to be friendlier to their children was seen as vital. It also came out that parents needed to relate with their children firmly and they were supposed to set behavioural boundaries for their children. For instance, it was believed that parents should be strict in stopping their children from watching pornography.

By and large, parents suggested that parenting seminars would help to arm them with knowledge and skills to improve communication with their children. Others suggested parents and children should be brought together during parenting seminars so that they learn how to respond to them to one another:

*“. . . if we go and tell our children that they told us this and that, the children will say that we are lying’ . . . children should be present in parenting seminars . . . because that is the way parents and children can learn how to respond to one another. . .”.*

## Discussion

This study examined the nature of parent-adolescent communication on SRH and how best it could be improved to promote healthy sexual behaviour among adolescents. Evidently, findings showed that a warm and loving relationship between parents and their children is foundational for good parent-adolescent communication. Similar to other studies [37, 38], the tendency for both male and female adolescents to communicate more openly and frequently with mothers than fathers even when these children lived with both biological parents was evident in this study. Contrary to studies which claim that parent-adolescent communication on sexuality in SSA is often negative, vague and based in authoritarian parenting styles [39–41], this study revealed the cordiality of the relationship between most mothers and their adolescents. This can partly be explained by the fact that young people spend more time with their mothers than their fathers. Unlike mothers, fathers were found to often be more strict, intimidating, unapproachable and/or unavailable. This was consistent with observations from studies in Tanzanian and South Africa which reported the tendency of fathers to meet adolescents’ inquisitiveness about sexuality with silence, warnings, threats and hostility [41, 42]. Similar to a study conducted in Ghana, this study found that fewer male adolescents were comfortable communicating with their parents and even fewer communicated with their fathers [43].

It was evident that male adolescents in general communicated less with anyone on issues of sex, relationships and condoms. When faced with questions about body changes, more female adolescents admitted that they talked with their mothers. Most discussions about SRH were found to be gendered with more caution on SRH being given to girls [44]. For cultural reasons, it was found that fathers were not expected to discuss puberty issues with their daughters and most discussions relating to female adolescents’ SRH appeared to take place in social places of women.

While a certain level of communication between parents and their young adolescents on SRH was evident, the discussions normally focused on HIV/AIDS, STIs and body changes. This is important since past research had indicated that increasing communication with other people about HIV and AIDS has a protective effect since it exposed adolescents to information and encouraged dialogue about risks and options [45]. Unfortunately, this study showed that both male and female adolescents were uncomfortable when it came to discussing, even with their mothers topics like dating and sex, which are a gateway to acquiring sexually transmitted infections.

It was found that parents stopped their adolescents from either talking about perceived embarrassing topics, refused to respond, or told them to wait and ask about these issues when they are older. This finding is consistent with past research which had found that in many sub-Saharan language dialects and Uganda in particular, there were no culturally appropriate words for many sexual terms or if they were there, they sounded vulgar [9, 19, 46]. Consequently, many young adolescents did not ask because they feared being misunderstood or perceived as being sexually active. Uniquely, this study established that when parents initiated discussions on SRH, it was often to warn young people about dangers of becoming sexually active at an early age, reinforcing the myth that condoms are not safe and dangers of befriending adolescents of the opposite sex, which was not different from what was found in two other studies in SSA [41, 47].

By and large, parents were reported to often initiate discussions on SRH issues at the onset of puberty or when they suspected that their children were starting to get involved with the opposite sex. In the view of adolescents, this was believed to be too late for such discussions. A common trigger for SRH discussions between young female adolescents and their parents was either the onset of menstruation or if a girl in the neighborhood got pregnant or died whilst trying to procure an abortion. For male adolescents, the discussions would often be triggered by parents suspecting that they had female friends or when they started coming home late at night. Similar to a study from Tanzania, the death of somebody in the neighborhood due to suspected-AIDS was found to be another trigger for parents to warn their children to be careful [42]. The cause of discomfort among parents was that if they started the discussion on SRH with their children too early, it could prompt their curiosity and drive them into high risk sexual behaviors.

Adolescents were found to largely get information about SRH issues from peers at schools and the mass media. It is conceivable that parents need to play a bigger role in helping adolescents to synthesize this information and to ensure that what adolescents got out of the exchange was accurate and factual. The downside of getting information from the media or other people is the possibility of getting misleading information. Indeed, discussions with adolescents revealed that they had a lot of misconceptions regarding consequences of delayed sexual debut and condoms. Secondly, adolescents trusted that their parents were more likely to give them accurate and good advice, a fact already established in another study [12]. It is therefore vital to improve discussions between adolescents and their primary caregivers about SRH issues.

Like elsewhere in most of SSA [48], the culturally sanctioned person tasked with discussing SRH issues with mostly adolescent girls was the paternal auntie (*Senga)*. Unfortunately, many *‘Sengas’* are reported to often have no formal training in SRH issues even though they are trusted to mostly prepare older girls betrothed for marriage. In Uganda’s contemporary society, the tendency was that the *Senga* role was getting commercialized and modern business-minded *Sengas* had emerged. Therefore, many parents in the study were reluctant to trust such *Sengas* with their children since they were no longer sure about the information passed on to their children.

### Methodological challenges

Methodological challenges associated with qualitative research need to be borne in mind when interpreting these findings. Recruitment of parents to participate in the study relied on school administrators as gate-keepers. It is possible that these gate-keepers introduced some level of selection bias. However, the effects of weakness were minimized if not avoided through use of purposeful sampling of parents selected by the gate-keepers to remain with those that were information-rich about the study issues.

Although the use of key informant interviews and focus group discussions was labour intensive and time-consuming, the approach enabled triangulation of data sources and improved the credibility of findings. A mix of such methods is known to ensure complementariness in data collection from individuals to enable exploration of issues in more depth. Although discussions on SRH issues tend to be private and closed in most African societies, this study reduced the effect of this challenge by making the discussion questions less personal and more about other people in the community.

During data collection, instances of misconstruing the researchers as designated representatives of a bilateral partner seeking to bolster the financial base of participating schools was noted. As such, the reception often extended was cordial and anticipatory in nature. Therefore, it is possible that participants’ responses could have been influenced by the desire to please the researchers for a secondary gain. The researchers had to assure study participants about who they were and the purpose of the research.

Lastly, it is not possible to make definitive generalizations from a study like this since the focus was in only four schools and given the study design, this is untenable. Future research can do better by enlisting more groups of study participants, more schools including some that are privately owned and use more methods in collecting and analysing the diverse data. By not selecting study participants from privately-run secondary schools because of differences in parent-adolescent interaction dynamics, we acknowledge this as a limitation.

### Implications

It is evident that parents need to understand that when young people ask questions about relationships, sex or condoms it is not necessarily because they are planning or are already engaged in relationships and sexual activity. They should instead encourage them to ask questions and seek clarifications, since doing so, the adolescents will be able to access more accurate information and dispel many of the misconceptions and incorrect information that surround sex and condoms.

The study showed that that perhaps, conversations on SRH issues between children and their parents could start when children become aware of their sexuality and body parts. It is evident that waiting for adolescence to start the discussions is too late, since there are many other sources of information on relationships and sex some of which is incorrect. Parents need to understand that by keeping silent, it does not necessarily mean that other agents of socialization are not communicating to their children. Depending on the source of information, young people could be exposed to wrong information. Consistent with the purpose of PREPARE project, information from this formative research was vital in understanding of context and ensuring that the intervention which was ultimately developed addressed barriers and facilitators to promotion of adolescent-parent communication on SRH issues *(findings from PREPARE intervention are in an upcoming paper reported elsewhere)*.

## Conclusions

For parents, it is clear that discussions of SRH issues with adolescents need to avoid being characterised by threats. Communication with adolescents should not necessarily be solely the domain of mothers. Fathers have a substantial role to play and they do not have to *‘come across’* as too strict, unapproachable, unavailable and too be busy. Other than the emphasis on education matters, male parents should become comfortable to discuss SRH issues as well. Parents need to realize that communication is at times compromised by their inability to initiate interesting topics, embarrassment, and fear.

Most potential life-saving messages need to be properly packaged while being delivered to children by parents. Currently, most messages are passed onto children through; fear-based messaging, intimidation, warnings and verbal abuse. There is need to develop a culturally appropriate language of communicating with adolescents and challenging their misconceptions associated with sexual maturation. The alternative is that in many languages, SRH issues are vulgarised and embarrassing.

Communication between parents and children should also be about topical SRH issues like consequences of sexually relating with the opposite sex, role of abstinence from sex, menstruation, HIV/AIDS, sexually transmitted infections, contraception, teenage pregnancy and its associated problems and bio-psychosocial changes associated with puberty. The bulk of parents need to be empowered and motivated to be bold and self-confident in communicating with their children. This is because many of them are uncomfortable talking to their own children about SRH issues like condom use. Parents should be attuned to teachable moments so as to seize the available moments to discuss with their adolescent children.

Parents should not wait to be caught off-guard by their children’s questions but can also initiate the discussions. They too do not have to be unnecessarily evasive. They should ask parents questions and seek clarifications. It was observed that the practice of some children saying that they have already been told about those *‘things’* by teachers at school is a strategy to block communication.

For in-school children, the school environment could be an outlet for improving parent-adolescent communication. Parents can conveniently be invited to schools to discuss their children’s issues. Teachers as educators should perform the role of parental training. Teachers should help parents to reflect on the quality, content, relevance and appropriateness of what they as teachers do concerning SRH issues at school to avoid a practice where parents assume that teachers do everything at school.
